# Preoperative albumin/globulin ratio has predictive value for patients with laryngeal squamous cell carcinoma

**DOI:** 10.18632/oncotarget.18443

**Published:** 2017-06-12

**Authors:** Wan-Zhi Chen, Shi-Tong Yu, Rong Xie, Yun-Xia Lv, De-Bin Xu, Ji-Chun Yu

**Affiliations:** ^1^ Department of Thyroid and Neck Surgery, The Second Affiliated Hospital of Nanchang University, Nanchang, Jiangxi, China; ^2^ Department of Otolaryngology, Head and Neck Surgery, Sun Yat-sen Memorial Hospital, Sun Yat-sen University, Guangzhou, Guangdong, China

**Keywords:** albumin/globulin ratio, laryngeal squamous cell carcinoma, prognostic marker

## Abstract

This study evaluated the predictive value of the preoperative albumin/globulin ratio (AGR) in laryngeal squamous cell carcinoma (LSCC) retrospectively, which has not been reported before. The current study enrolled 241 newly diagnosed LSCC patients in the Second Affiliated Hospital of Nanchang University between January 2005 and December 2010. The optimal AGR cut-off value for overall survival (OS) was determined to be 1.28. Univariate survival analysis identified sex, low AGR, T classification, histological grade and nodal metastasis as factors associated with poor OS. Additionally, a low AGR, T classification, nodal metastasis, and histological grade were associated with poor disease-free survival (DFS) in LSCC patients. In multivariate survival analysis, nodal metastasis and a low AGR remained significant for OS and DFS. Our preliminary study revealed that low preoperative AGR could serve as a valuable and easily-assessed blood-based indicator to predict the prognosis of LSCC patients.

## INTRODUCTION

Laryngeal cancer is responsible for 13430 new cases and 3620 cancer-related deaths each year in the US and 26400 new cases and 14500 cancer-related deaths annually in China [1, 2]. The most common pathological subtype is laryngeal squamous cell carcinoma (LSCC). Although significant progress has been made both in the diagnosis and treatment of LSCC in the past few decades, the mechanism and prognosis are still uncertain. Thus, identifying a new prognostic marker is urgent for better assessing patients at the time of diagnosis and for optimizing therapy.

Current methodologies for evaluating the prognosis of LSCC patients are mainly based on the tumor-node-metastasis (TNM) staging system, which can be used both to determine the clinical treatment strategy and to predict their outcome. Additionally, several inflammation-associated biomarkers, including the neutrophil to lymphocyte ratio (NLR), platelet to lymphocyte ratio (PLR) and C-reactive protein to albumin ratio (CAR), have been reported to have prognostic significance in LSCC [3–7].

Albumin (ALB) and globulin (GLB) are two important components of serum proteins and have been proven to be involved in the systemic inflammation. A low serum ALB reflects a poor nutritional status and has been proven to be an independent predictor of poor survival in various cancers, including lung cancer, nasopharyngeal cancer, and breast cancer [8, 9]. Moreover, an increased GLB level may reflect a chronic inflammatory response and cumulative exposure to various inflammatory cytokines [10]. Thus, the cumulative effect of both ALB and GLB may provide good prognostic value for cancer patients. However, no studies have reported this cumulative effect among LSCC patients. Therefore, we conducted the current study to evaluate the predictive value of the pretreatment ALB/GLB ratio (AGR) in patients with LSCC.

## RESULTS

### Patient characteristics

**Figure 1:**
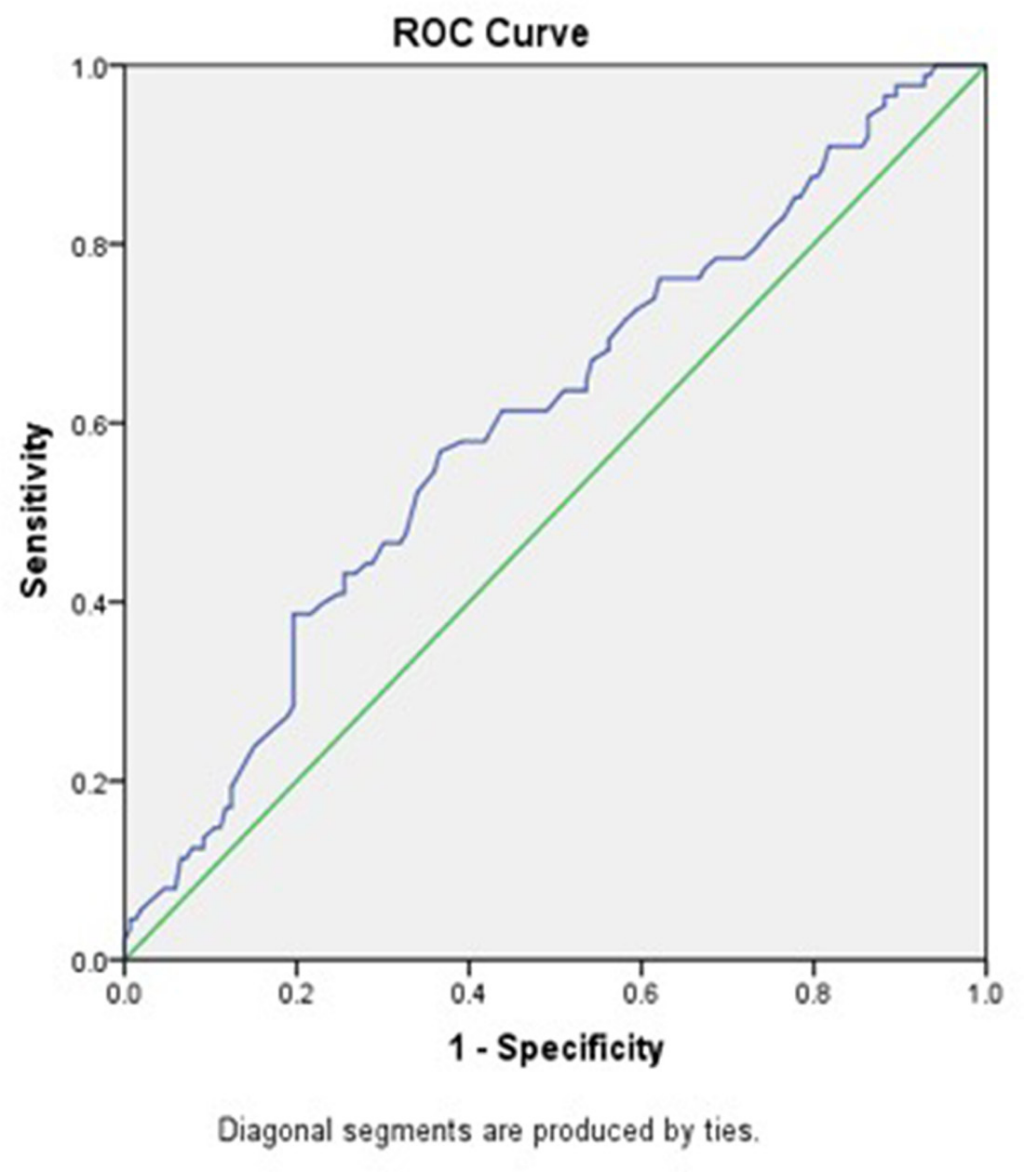
ROC curve analysis of the AGR of the survival status in the 241 patients with LSCC Notes: The optimal cut-off value was 1.28 according to ROC analysis (area 0.603, *P*=0.006). The green line is the reference line. The blue line is the curve for AGR. AGR, albumin/globulin ratio; LSCC, laryngeal squamous cell carcinoma; ROC, receiver operating characteristic.

In total, 223 men and 18 women with a median age of 55 years (interquartile range, 48-67). Among these patients, according to the 7^th^ AJCC/UICC TNM staging system, 78 (32.4%) patients were in stage I, 69 (28.6%) patients were in stage II, 61 (25.3%) patients were in stage III, and 33 (13.7%) patients were in stage IV. Forty-four patients chose to undergo postoperative radiotherapy, and 47 patients underwent postoperative chemo-radiotherapy at the Department of Radiotherapy of the Second Affiliated Hospital of Nanchang University. The median value of ALB was 39.70 g/L (interquartile range, 36.55-42.10), and the median value of GLB was 30.00 g/L (interquartile range, 25.10-37.60). The median value of AGR was 1.31, and the optimal cut-off value for OS was 1.28 (AUC 0.603, *P*=0.006) according to he ROC curve plotted in Figure 1.

The 241 patients enrolled were then divided into 2 groups based on the optimal cut-off value, and their clinicopathological features are compared and presented in Table 1. A lower AGR was shown to have an association with the T classification (*P*=0.01), nodal metastasis (*P*=0.01), and recurrence of disease (*P*=0.03). No other factors were found to be significantly associated with this ratio.

**Table 1:** Patient demographics and clinical characteristics

Characteristics	Total (n,%)	ALB/GLB ratio		*P*
		<1.28 (n,%)	≥1.28 (n,%)	
Total	241	90	151	
Age, years				0.87
≤60	107(44.4%)	38(42.2%)	69(45.7%)	
>60	134(55.6%)	52(57.8%)	82(54.3%)	
Sex				0.06
Male	223(92.5%)	87 (96.7%)	136(90.1%)	
Female	18(7.5%)	3(3.3%)	15(9.9%)	
Smoking status				0.57
Non or ex-smokers	96(39.8%)	37(41.1%)	59(39.1%)	
Smokers	145(60.1%)	47(58.9%)	92(60.9%)	
Cancer sites				0.51
Glottis	152(63.1%)	54(60.0%)	98(64.9%)	
Supraglottic	52(21.6%)	23(25.6%)	29(19.2%)	
Subglottic	37(15.3%)	13(14.4%)	24(15.9%)	
T classification				0.01*
T1+2	156(64.7%)	49(54.4%)	107(70.9%)	
T3+4	85(35.3%)	41(45.6%)	44(29.1%)	
Nodal classification				0.01*
N0	160(66.3%)	51(56.7%)	109(72.2%)	
N+	81(32.7%)	39(43.3%)	42(27.8%)	
Histological grade				0.58
Low	107(44.4%)	42(46.7%)	65(43.0%)	
High	134(55.6%)	48(53.3%)	86(56.0%)	
Recurrence				0.03*
Yes	88(36.5%)	42(46.7%)	49(32.5%)	
No	153(63.5%)	48(53.3%)	102(67.5%)	

* means statistical significance.

ALB/GLB, albumin/globulin.

### Survival analysis

**Figure 2:**
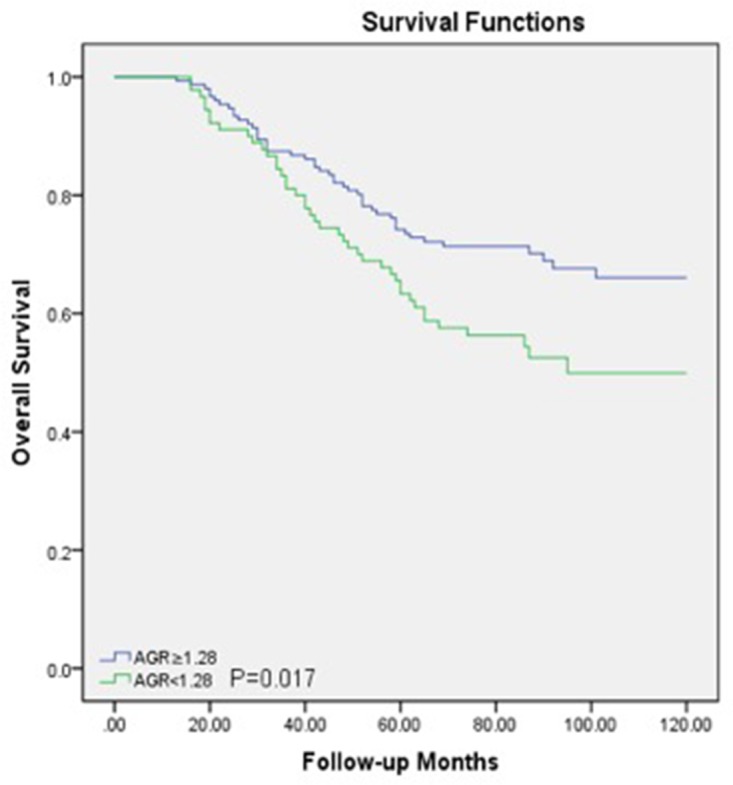
Overall survival curve for the 241 LSCC patients stratified by AGR (*P*=0.017) AGR, albumin/globulin ratio; LSCC, laryngeal squamous cell carcinoma.

**Figure 3:**
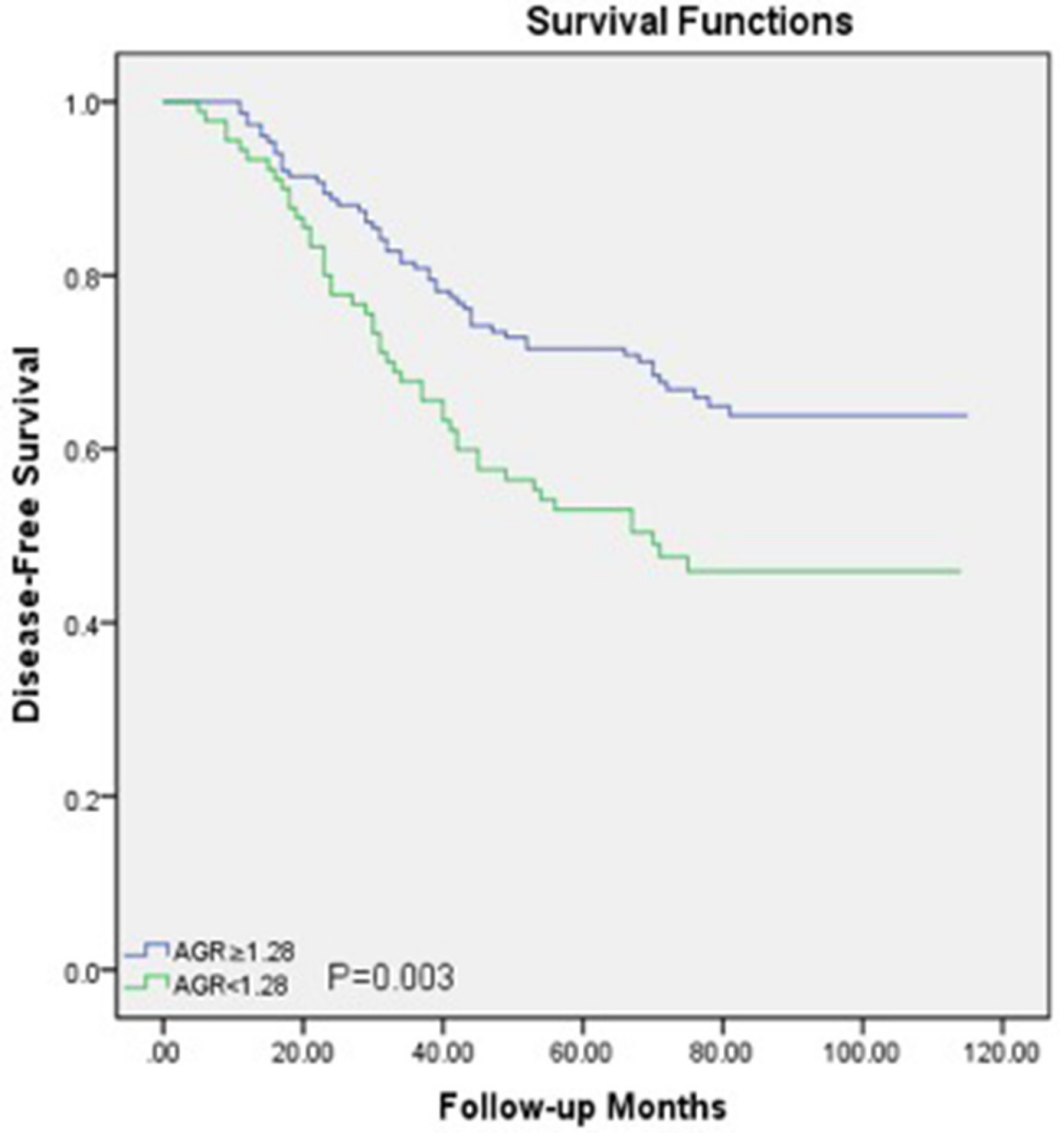
Disease-free survival curve for the 241 LSCC patients stratified by AGR (*P*=0.003) AGR, albumin/globulin ratio; LSCC, laryngeal squamous cell carcinoma.

Within a median 73 (62-89) months of follow-up, 88(36.5%) patients died from LSCC and 99 (41.1%) patients experienced recurrences of LSCC before the end of the follow-up period. In the Cox univariate models (Table 2), poor OS outcomes were significantly associated with sex (*P*=0.04), T classification (*P*=0.04), nodal metastasis (*P*=0.001), low histological grade (*P*=0.03) and lower AGR (*P*<0.05). The 5-year OS rate was better in patients with a higher AGR (≥1.28) compared to those with a lower AGR (<1.28) (Figure 2). For DFS, several clinicopathological indices, including the T classification (*P*=0.03), nodal metastasis (*P*=0.001), low histological grade (*P*=0.03), and lower AGR (*P*=0.006), were found to be significantly associated as well. Additionally, patients with a higher AGR were enjoyed a higher 5-year DFS rate compared to those with lower AGR values (Figure 3).

**Table 2:** Univariate Cox regression analysis for overall survival and disease-free survival in patients with laryngeal squamous cell carcinoma

Variables	No. of patients	OS		DFS	
		HR(95% CI)	*p* value	HR(95% CI)	*p* value
Sex			0.04*		0.14
Female	18	0.22(0.06-0.90)		0.44(0.15-1.2)	
Male	223	1		1	
Age, y			0.71		0.60
≤60	107	1		1	
>60	134	1.08(0.71-1.64)		1.12(0.75-1.66)	
Smoking status			0.54		0.43
Non or ex-smokers	96	1		1	
Smokers	145	1.28(0.87-1.79)		1.29(0.79-2.16)	
Cancer sites			0.73		0.56
Glottic	152	1		1	
Supraglottic	52	1.12(0.78-1.86)		1.24(0.46-1.89)	
Subglottic	37	1.27(0.86-2.03)		1.36(0.55-2.27)	
T classification			0.04*		0.03*
T1+2	156	1		1	
T3+4	85	1.55(1.10-2.23)		1.89(1.26-2.57)	
Nodal classification			0.001*		0.001*
N0	160	1		1	
N+	81	2.09(1.38-3.17)		1.77(1.21-2.54)	
Histological grade			0.03*		0.02*
Low	107	1		1	
High	134	1.32(1.16-2.04)		1.47(1.21-2.10)	
ALB/GLB Ratio			0.02*		0.006*
<1.28	90	1.67(1.10-2.54)		1.80(1.21-2.67)	
≥1.28	151	1		1	

* means statistical significance.

ALB/GLB, albumin/globulin; OS, overall survival; DFS, disease-free survival.

Multivariate analysis (Table 3) suggested that a lower AGR (hazard ratio [HR] = 1.35, 95% confidence interval [95%CI] = 1.10-2.12, *P*=0.04), and nodal metastasis (HR[95%CI] = 1.63[1.13-2.51], *P*=0.03) remained significantly associated with poor OS. For DFS, nodal metastasis (HR[95%CI] = 1.32[1.04-1.99], P=0.04), and a lower AGR (HR[95%CI] = 1.56 [1.08-2.71], P=0.008) remained significance. However, sex as well as the T classification and histological grade showed no significance in multivariate analysis.

**Table 3:** Multivariate Cox regression analysis for overall survival and disease-free survival in patients with laryngeal squamous cell carcinoma

Variables	No. of patients	OS		DFS	
		HR(95% CI)	*p* value	HR(95% CI)	*p* value
Sex		0.44(0.08-1.31)	0.36	-	-
Female	18				
Male	223				
T classification		1.07(0.79-1.42)	0.23	1.20(0.89-1.83)	0.17
T1+2	156				
T3+4	85				
Nodal classification		1.63(1.13-2.51)	0.03*	1.32(1.04-1.99)	0.04*
N0	160				
N+	81				
Histological grade		1.17(0.66-1.63)	0.49	1.33(0.82-1.65)	0.25
Low	107				
High	134				
ALB/GLB Ratio		1.35(1.10-2.12)	0.04*	1.56(1.08-2.71)	0.008*
<1.28	90				
≥1.28	151				

* means statistical significance.

ALB/GLB, albumin/globulin; OS, overall survival; DFS, disease-free survival.

## DISCUSSION

In this study, we identified preoperative AGR as an independent prognostic factor in LSCC patients. A low AGR value was associated with the T classification, nodal metastasis, and recurrence. In multivariate Cox regression models, we found that AGR was a prognostic factor for DFS and OS in LSCC patients. To the best of our knowledge, this is the first study to report the prognostic value of preoperative AGR in LSCC patients.

Preoperative systemic inflammation plays a significant role in various types of cancers [11] and may lead to tumor progression and metastasis by damaging the immune system and changing the tumor microenvironment, thus resulting in poor survival [9, 12–14]. Recently, several inflammation-based markers, including NLR, PLR and CAR, have been established to predict the survival in LSCC [3–7]. Tu XP et al. [4] determined a NLR cut-off value of 2.17, and reported that a higher NLR was associated with OS and DFS. Wong BY et al. [6] reviewed 140 patients and found that an elevated NLR was significantly associated with an advanced disease stage and poor OS. In a report by Wang J et al. [5], the optimal cut-off value for NLR and PLR were identified as 2.79 and 112, respectively. In a multivariate Cox analysis, they found that a higher pretreatment PLR was associated with OS and DFS. ALB and GLB are two important components of systemic inflammation, and the combination of these two markers (AGR) has been reported to be significant and validated in several types of cancers, including nasopharyngeal carcinoma, small-cell lung cancer and hepatocellular carcinoma [15–18].

There may be several explanations of our findings. First, albumin is a major protective component can against carcinogenesis by nitrosamine and aflatoxin and can stabilize DNA replication and cell growth, and buffer sex hormone homeostasis to prevent sex hormone-induced cancer [19]. Additionally, the growth of many cancer cell lines can be inhibited by high concentrations of albumin [19–21]. Notably, the generation of albumin can be suppressed by malnutrition and inflammation, which are triggers for many cancers [8]. Interleukin-6(IL-6), for instance, can promote the synthesis of acute-phase reaction proteins in the liver and reduce the synthesis of albumin by liver cells [10, 22, 23]. Secondly, a low ALB level may reflect malnutrition among cancer patients. In this circumstance, patient immune systems were relatively vulnerable, including cellular and humoral immunity and phagocyte function, which facilitated infection and compromised the responses to treatment [24]. Thirdly, rising levels of globulins may reflect an inflammatory state marked by the accumulation of acute-phase proteins, immunoglobulins and other serum proteins. Furthermore, higher levels of alpha and gamma globulins were reported to be prognostic indicators for lung cancer patients [25]. As mentioned above, the inflammatory state may play an important role in the carcinogenesis of LSCC.

In the current study, the optimal cut-off value for AGR was 1.28, which was determined by ROC analysis. This value was close to the optimal cut-off value (1.3) reported in studies on esophageal squamous cell carcinoma [26]. The use of diverse AGR cut-off values in a diverse cohort of LSCC patients may lead to varied survival results. Therefore, optimal and generalized AGR thresholds for LSCC patients should be determined in the future with a better-designed study and a larger cohort.

The current study had several limitations. Firstly, it was a retrospective-design single center study with a relatively short follow-up and small sample size, which may lead to a poorly generalizable result. Second, many reported prognostic indices, including PLR and NLR, have not been investigated and compared with AGR, and other inflammatory markers, including interleukin and tumor necrosis factor, were unavailable. Third, the AUC is relatively low. Fourth, some patients were excluded from this study due to loss to follow-up, which may reflect a selection bias. However, our preliminary findings suggest that AGR may be an effective prognostic marker for LSCC patients. Therefore, a larger LSCC cohort with well-documented medical history as well as other prognostic markers should be investigated in the future to validate our findings.

## MATERIALS AND METHODS

### Patients

Between January 2005 and December 2010, this study evaluated a consecutive cohort of 437 newly diagnosed LSCC patients who underwent surgery at the Department of Thyroid and Neck Surgery at the Second Affiliated Hospital of Nanchang University. The exclusion criteria were as follows: patients who underwent preoperative chemotherapy and/or radiotherapy, patients with synchronous cancer, a history of cancer, the presence of infection or inflammatory conditions, and those without pretreatment blood tests. Finally, 241 patients were enrolled into the final analysis.

### Treatment and follow-up

The treatment protocol included standard laryngectomy (+/- neck dissection) and postoperative radio-/chemotherapy according to the National Comprehensive Cancer Network guidelines. Postoperative radiotherapy or chemoradiotherapy was performed for patients with adverse features (e.g., extracapsular node spread, positive margins, pT4 primary, N2/3 nodal disease, and perineural invasion). All patients were followed-up postoperatively every 3 months for the first 2 years, and every 6 months thereafter for up to 5 years or until death. Postoperative laryngoscopy and neck ultrasound/computed tomography (CT) were used routinely. Recurrence was defined as any newly detected mass on image examinations which was histologically confirmed by ultrasound-guided biopsy or surgery.

### Data collection

All patients were agreed to reviewed of their medical records, and the ethics committee of the Second Affiliated Hospital of Nanchang University approved this study. The American Joint Committee on Cancer (AJCC)/Union for International Cancer Control (UICC) TNM staging system (7^th^ edition) was to classify disease stage. All patients enrolled into this study had blood samples taken before any therapeutic intervention. All ALB and GLB analyses were performed at the laboratories of the Second Affiliated Hospital of Nanchang University using a standard methodology. The GLB was calculated as total serum protein-ALB. The ALB/GLB ratio was calculated as serum ALB divided by serum GLB level.

### Statistical analyses

Continuous variables were expressed as median with interquartile ranges. The Chi-square or Mann-Whitney U test was used to compare the descriptive statistics of the patient demographic and clinical characteristics (including gender, age, smoking status, cancer sites, T stage, nodal status and survival/recurrence status).

Receiver operating characteristic (ROC) curves were drawn to determine the optimal cut-off value as well as the area under the curve (AUC) for overall survival (OS). The Kaplan-Meier method was used to analyze survival curves. The log-rank test was used to evaluate the differences in survival rates among different groups. Multivariate analysis using the Cox proportional hazards model was performed to analyze prognostic factors associated with OS and disease-free survival (DFS) based on the factors proven to be significant in the univariate analysis. A two-sided P value less than 0.05 was considered statistically significant. All analyses were performed using SPSS v22.0 (IBM Corporation, Armonk, New York, USA).

## CONCLUSION

Our preliminary report revealed that a low AGR value was associated with the T classification, nodal metastasis, and recurrence in LSCC patients. Additionally, AGR was found to be an independent prognostic marker for both OS and DFS in this study. Our finding suggests that preoperative AGR, as an easily-accessed, blood-based marker, combined with the established TNM staging system can help doctors to predict treatment outcomes better and may guide individualized treatment. Further prospective case-controlled studies with a larger cohort enrolled with various prognostic markers, including AGR, NLR, PLR, as well as other indices, should be conducted to validate our findings.
